# Systematic exploration of network pharmacology, *in silico* modeling and pharmacokinetic profiling for vitamin E in polycystic ovarian syndrome

**DOI:** 10.2144/fsoa-2023-0245

**Published:** 2024-05-15

**Authors:** Rukaiah Fatma Begum, Sumithra Mohan

**Affiliations:** 1 Department of Pharmacology, SRM College of Pharmacy, SRM Institute of Science & Technology, Kattankulathur-603203, Chengalpattu, Tamil Nadu, India

**Keywords:** computational approach, *in silico*, network pharmacology, polycystic ovarian syndrome, vitamin E

## Abstract

**Aim:** This study seeks to explore the possibility of using vitamin E to alleviate the symptoms of polycystic ovarian syndrome (PCOS). **Methods:** Various computational methods were employed, including network pharmacology utilizing a compound-target-pathway approach, Swiss ADME, OSIRIS^®^ property explorer, pkCSM, PASS online web resource and MOLINSPIRATION^®^ software. In addition, *in silico* analysis of vitamin E was performed with ten receptors. **Results & discussion:** Our findings highlight the diverse potential of vitamin E in alleviating PCOS. The observed influence on hormones is in line with existing PCOS theories regarding cyst development, further enhancing the therapeutic promise of vitamin E. **Conclusion:** In conclusion, our computational analysis indicates that vitamin E shows potential as a therapeutic agent for alleviating PCOS in adolescents.

## Plain language summary

Polycystic ovarian syndrome (PCOS) is a common condition that affects many girls and young women during their reproductive years. PCOS can lead to problems with the menstrual cycle, difficulties with blood sugar control and sometimes infertility. Unfortunately, there's no specific treatment for PCOS yet. This study looked at whether vitamin E might be helpful in treating PCOS in young women. To find out, the researchers used advanced computer-based techniques and analyzed how vitamin E interacts with various parts of the body, including proteins and hormones. Vitamin E appears to influence hormones that play a role in PCOS and cyst formation. It may also have anti-inflammatory properties that could help reduce the symptoms of PCOS. In short, this study suggests that vitamin E might be a valuable option for treating PCOS in adolescents. However, further research and clinical trials are needed to confirm these findings before it can become a standard treatment. This research opens up new possibilities for finding effective solutions for PCOS, which can greatly improve the quality of life for many young women.

## Abstract (plain language summary)

Network pharmacology studies have significantly contributed to unraveling the intricate molecular mechanisms associated with PCOS and vitamin E, shedding light on its impact on signaling pathways.

The docking scores of PPAR and estrogen receptors, reaching as high as -9.3, -7.8 and -7.5 Kcal/mol, underscore the potential of vitamin E to exert biological activity in the context of PCOS.

ADME studies have elucidated the inhibitory effects of vitamin E on critical cytochrome P450 enzymes, specifically CYP3A4 and CYP2C19, further emphasizing its promising pharmacological potential.

Vitamin E demonstrates a favorable safety profile, with no observed mutagenicity, tumorigenicity, irritancy, or adverse effects on reproduction, substantiating its suitability for therapeutic applications.

Polycystic ovarian syndrome (PCOS) is a widely prevalent and intricate endocrine disorder impacting a substantial number (5–15%) of women across the globe [1]. It is identified by a mix of irregularities in hormone levels and disruptions in metabolism, which can lead to various health-related issues. PCOS is renowned for its varied array of symptoms and its potential to affect numerous facets of a woman's life, encompassing fertility, menstrual patterns and overall health and well-being [2]. Recognizing the significance of comprehending PCOS is crucial, as it not only exerts an influence on a woman's reproductive health but also carries implications for her long-term well-being. This has rendered PCOS a subject of considerable interest and investigation within the domains of gynecology, endocrinology and reproductive medicine [3,4]. PCOS is a multifaceted disorder characterized by an array of symptoms and possible complications, necessitating treatment plans that target particular symptoms. These approaches can encompass lifestyle adjustments, pharmaceutical interventions and, on occasion, surgical procedures. Below are some typical methods for addressing PCOS [5,6]. The emergence of PCOS is influenced by a complex interplay of an individual's genetic and environmental factors. Genetic elements encompass a family history of PCOS among immediate relatives, early onset of sexual maturity and preterm fetal development. Environmental factors encompass a sedentary lifestyle, consumption of high-fat, high-sugar and high-salt junk food, exposure to advanced glycation end products (AGEs) and obesity [7]. The management of PCOS is diverse and personalized, taking into account the unique requirements and objectives of every patient. Moreover, except for estrogen-progestin combinations, there are no medications authorized for the explicit purpose of addressing metabolic irregularities and excess androgens. Consequently, the majority of drugs are prescribed for uses not officially approved [8].

In adolescents, the diagnosis of PCOS is a subject of debate, leading to inconsistency in both evaluation and treatment. Unfortunately, the requirements of young women with PCOS are not being sufficiently fulfilled [9]. Medications such as metformin, oral contraceptives and myoinositol can provide assistance to adolescents dealing with PCOS [10]. It's important to note that oral contraceptives should not be prescribed to teenagers for durations exceeding 3 years [11]. Nutritional research emphasizes the significance of dietary adjustments in managing this condition. Girls with PCOS generally align with recommended macronutrient distribution ranges for carbohydrates, proteins and fats [12]. However, there were no observed distinctions in dietary quality or food intake between women with and without PCOS. This highlights the need for novel guidelines that advocate for the integration of healthy lifestyle modifications as a key element of treatment, considering the absence of a specific and deficient dietary target for interventions [13].

In this introductory discourse, we shall delve into the essential attributes, causative factors, diagnostic approaches and prospective therapeutic interventions associated with PCOS, emphasizing the critical need to address this condition for the betterment of women's health. Currently, owing to the unclear origin of PCOS, there is no established and efficacious treatment regimen. Clinical practice primarily relies on managing symptoms and ongoing health maintenance is essential.

Vitamin E, a type of antioxidant vitamin that dissolves in fats, has attracted attention for its potential role in addressing certain symptoms linked to PCOS. Vitamin E is known for its ability to neutralize harmful free radicals in the body. Oxidative stress, which occurs when there is an imbalance between free radicals and antioxidants, is associated with the pathophysiology of PCOS. Elevated oxidative stress can exacerbate inflammation and insulin resistance, common features of PCOS. Vitamin E's antioxidant properties may help reduce oxidative stress and mitigate these effects [14]. One hallmark of PCOS is insulin resistance, a condition where the body's cells become less responsive to insulin, leading to elevated blood sugar levels. Emerging evidence suggests that vitamin E may improve insulin sensitivity in PCOS patients, potentially by reducing oxidative stress and inflammation. Improved insulin sensitivity can help manage glucose levels and decrease the risk of type 2 diabetes, which is often associated with PCOS [15,16]. Vitamin E's anti-inflammatory properties may help reduce inflammation in PCOS patients, potentially alleviating some of the associated symptoms [17]. For women with PCOS seeking to conceive, vitamin E supplementation may offer potential benefits. By addressing insulin resistance and hormone imbalances, vitamin E could enhance fertility and increase the chances of successful pregnancy [18,19].

Through computational methods, this study explores the hormonal and anti-inflammatory attributes of vitamin E. The study utilized network pharmacology, a method that explores the connections between diseases, genes and potential treatments. This approach aimed to uncover the molecular foundations of drug actions and biological systems, encompassing human organs, diseases, metabolic pathways and target proteins [20,21]. Additionally, the study involved analyzing and summarizing pharmacokinetic parameters, potential adverse effects and toxicity assessments. Various online servers and web resources were employed during the study. The final conclusions were based on a combination of online and offline technologies, offering insights into different pharmacological aspects and parameters. The utilization of *in silico* methods to assess the pharmacology of drug candidates offers a comprehensive understanding of factors like absorption, distribution, metabolism, excretion and toxicity [^22–24^]. Consequently, it allows for the improvement of new compounds with a strong attraction to the specific target region.

## Methodology

### Screening of disease targets for PCOS & vitamin E

We conducted searches using the terms ‘PCOS’ and ‘polycystic ovarian syndrome’ in online gene databases, including DisGeNet (https://www.disgenet.org/), GeneCard (https://www.genecards.org/). The gene data obtained from these searches were collated and duplicates were removed. Following data normalization using the UniProt database, we identified relevant targets for PCOS treatment. Additionally, we conducted searches for the keywords ‘vitamin E’ and ‘alpha-tocopherol’ in Swiss Target Prediction (http://swisstargetprediction.ch) to identify genes associated with the compound.

### Constructing PPI networks & screening for crucial targets

Protein-protein interactions (PPIs) are of utmost importance in a diverse array of cellular processes and are indispensable for the functioning of living organisms. Grasping the intricacies of PPIs is critical for gaining insights into the complexities of molecular biology, cellular signaling and the mechanisms underpinning various physiological and pathological processes. To construct a PPI network involving overlapping targets, the STRING database (https://string-db.org/) was utilized, with a focus on ‘homo sapiens’ as the species. Subsequently, the PPI network was generated using Cytoscape_V3.9.1. An in-depth analysis of the network was conducted through the Cytoscape network analyzer tool, which allowed the determination of degree values and accuracy. Following this analysis, the top ten targets with the highest degree scores were selected for subsequent *in silico* molecular docking.

### Enrichment analysis by GO & KEGG pathway

The outcomes of pathway enrichment analysis using Gene Ontology (GO) and Kyoto Encyclopedia of Genes and Genomes (KEGG) were utilized on the STRING online database (https://string-db.org/) to label and categorize the 25 common targets. Subsequently, with a p-value cutoff of 0.05, we gathered and assessed the data employing Rstudio 3.6.3 (Bioconductor clusterProfiler).

### *In silico* molecular docking

According to the degree analysis and enrichment results of the GO and KEGG, we selected the top ten proteins which was further taken to molecular docking with vitamin E. The crystal structures of receptors were obtained from PDB protein database (http://www.rcsb.org/) based on the sequence alignment and activity. The compound structure was obtained from the pubchem (https://pubchem.ncbi.nlm.nih.gov/) in SDF format and was converted to PDB by Open Babel GUI 2.4.1 for docking interpretation. Molecular docking was performed out further with AutoDock Vina tool 1.5.6. After the removal of water molecules and the addition of hydrogen atoms to improve the stability of amino acid residues in their various forms, the refined protein structure was further optimized. This corrected protein structure was then saved as a ‘protein.pdb’ file, which was used in all subsequent docking experiments. By removing water and ligands connected by covalent bonds, adjustments were made to the bond order. This process included the application of charge and protonation states within the molecular mechanics force field to achieve energy minimization. AutoDock Vina software was used to calculate Gasteiger charges and evaluate the ligand's rotatable bonds. This process generated various conformations for docking. For each receptor, receptor grids were created by defining grid points (size x,y,z with 40 × 40 × 40, respectively) and the grid boxes were built using these reference axes with the exhaustiveness of 8 [25,26]. The binding affinity between the molecule and proteins is predicted based on docking minimum free energy. The results were saved in pdbqt file which was visualized and presented by Biovia Discovery Studio 2021 client.

### Estimation of pharmacokinetic profile

The canonical SMILES notation for vitamin E was obtained from the PubChem web server and subjected to pharmacokinetic assessments using pkCSM and Swiss ADME [27]. These assessments, based on the canonical SMILES, provide valuable information about the drug's absorption, distribution, metabolism, excretion and toxicity. pkCSM, a reliable online tool, is well-suited for exploring medication pharmacokinetics and has been previously utilized to analyze the physicochemical properties and analgesic efficacy of N-benzoyl thiourea derivatives [28].

### Toxicity prediction

It is crucial to carry out toxicological evaluations when withdrawing a drug to avoid potential adverse effects, including organ system failure or harm. The compounds' chemical composition was compared with PubChem data to determine their toxicity profile, using the OSIRIS^®^ property explorer program. This assessment utilizes a color-coded scale to measure characteristics such as tumorigenicity, mutagenicity, irritant potential, reproductive effects and drug suitability [29,30]. It also produces values for factors like Topological Polar Surface Area (TPSA), drug similarity and anf overall drug rating. Here, the color green represents a low risk of toxicity, yellow indicates a moderate risk of toxicity and red signifies a high risk of toxicity

### Biological activity prediction using PASS online tool

The online tool Prediction of Activity Spectra for Substances (PASS) was employed to predict the biological activity of the drugs. By analyzing the structural formula of the compound the tool projects their biological activity spectrum, emphasizing the link between structure and function [31,32]. Biological activity is a crucial aspect of the characteristics of chemical compounds, as it determines their utility in medical applications [33]. Additionally, the PASS ONLINE website resource was used to anticipate the potential side effects of these drugs. The PASS online software is employed for assessing biologically active substances, offering predictions for over a huge number of drug pharmacological actions and biochemical mechanisms, all according to the structural formula of the substances. This predictive capability can aid in the identification of novel pharmacological targets [34].

### Molecular property & bioactivity score assessment

Computational testing of the chosen drugs was carried out using MOLINSPIRATION^®^ cheminformatics software to assess drug resemblance and forecast bioactivity [35]. The bioactivity score, which falls within the range of -0.50–0.00, indicates moderate activity, with a score of 0.00 signifying inactivity. Medications with a bioactivity grade below 0.00 are considered to possess optimal biological activity.

## Results

### Target screening & prediction of PCOS & vitamin E

**Figure 1.**
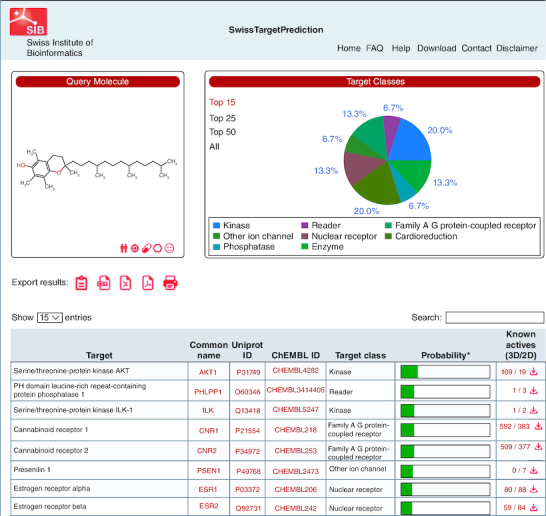
Swiss target prediction for vitamin E.

**Figure 2.**
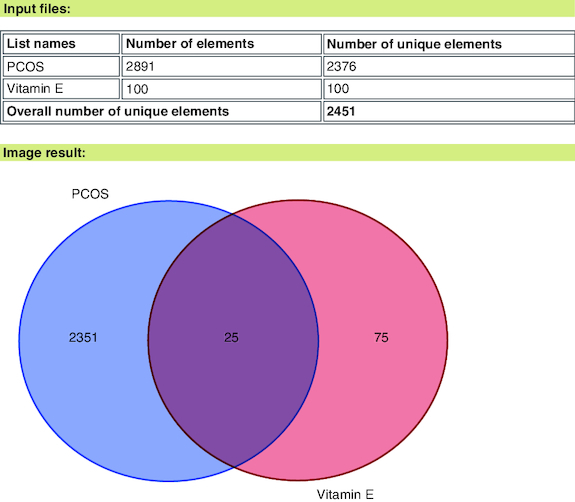
Venn diagram.

A comprehensive screening process initially identified 2891 proteins associated with PCOS-related target genes, sourced from the GeneCards and DisGeNET databases, after eliminating duplicates. In parallel, 100 proteins were identified as vitamin E-related target genes using Swiss target prediction (Figure 1). These two sets were consequently merged, resulting in the identification of a final set of 25 common target genes, as depicted in the Venn diagram (Figure 2). Subsequently, these 25 common target genes were then subjected to further analysis in PPI studies.

### Constructing of protein-protein interaction (PPI) network

**Figure 3.**
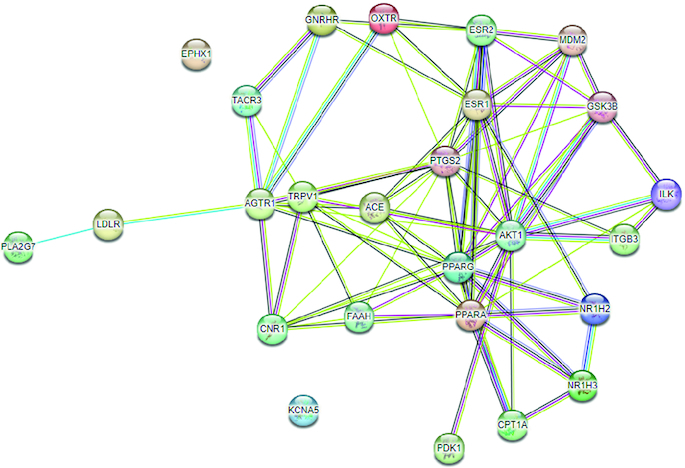
Interactions between proteins encoded by genes.

The target genes were inputted into the STRING online database to create a PPI network consisting of 26 nodes (genes), resulting in the identification of 80 edges (interactions), as depicted in Figure 3. To visualize the protein interactions within the PPI network, Cytoscape v_3.9.1 was employed. Following that, the network analyzer was employed to determine the degree values of the shared target genes, a crucial metric for assessing network nodes. According to these degree values, the top ten targets were *AKT1, PTGS2, PPARG, PPARA, ESR1, LDLR, GSK3B, CNR1, ACE* and *ESR2*, with degrees of 17, 14, 13, 12, 10, 9, 9, 8, 7 and 6, respectively. Additionally, topological parameters such as betweeness centrality and closeness centrality was determined for all 25 common target genes (Table 1). These targets serve as central hubs within the PPI network, connecting and influencing various nodes.

**Table 1.** Network topological parameters of top ten target genes.

Target	Degree	Betweenness centrality	Closeness centrality
*AKT1*	17	0.245475	0.81481
*PTGS2*	14	0.126482	0.70968
*PPARG*	13	0.100128	0.70968
*PPARA*	12	0.080057	0.68750
*ESR1*	10	0.069223	0.61111
*LDLR*	9	0.105911	0.59459
*GSK3B*	9	0.049476	0.62857
*CNR1*	8	0.042888	0.59459
*ACE*	7	0.014694	0.59459
*ESR2*	6	0.017711	0.55000

### Exploration of KEGG pathways & GO function enrichment

**Figure 4.**
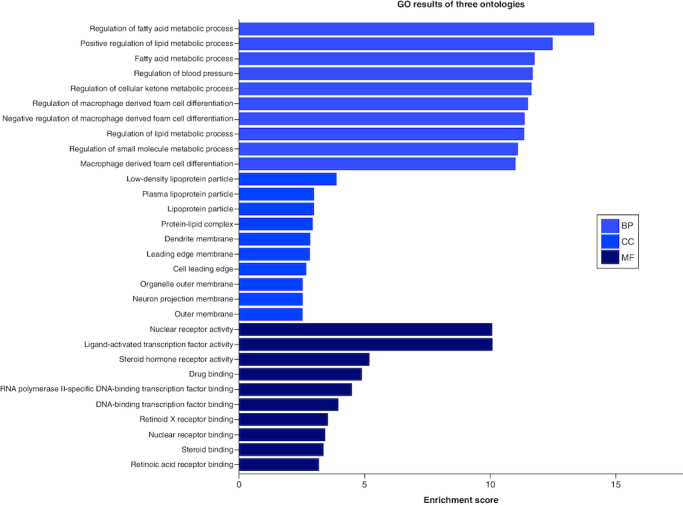
Gene ontology enrichment analysis (BP-biological process, CC-cellular components and MF molecular function).

**Figure 5.**
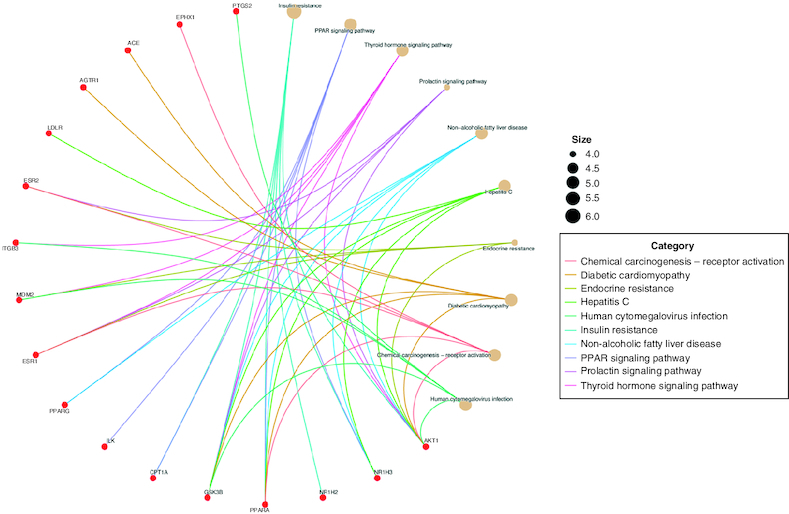
KEGG enrichment analyses.

We conducted GO enrichment analysis and the top ten results for biological processes (BP), molecular functions (MFs) and cellular components (CCs) are presented in Figure 4. These results indicate that the biological processes involve the regulation of fatty acids and lipid metabolic processes, blood pressure regulation, cellular ketone metabolic processes and cell differentiation. In terms of molecular functions relevant to drug-disease interactions, the analysis revealed activities such as nuclear receptor function, steroid hormone receptor activity, drug binding and RNA and DNA transcription factor binding. The KEGG enrichment analysis was used to identify the associated pathways for vitamin E. We identified 154 signaling pathways, with the top ten mentioned in Figure 5. Notably, the analysis emphasized pathways like insulin resistance, Peroxisome Proliferator-Activated Receptors (PPAR) [36], thyroid hormone signaling and the prolactin signaling pathway.

### Compound target disease network

**Figure 6.**
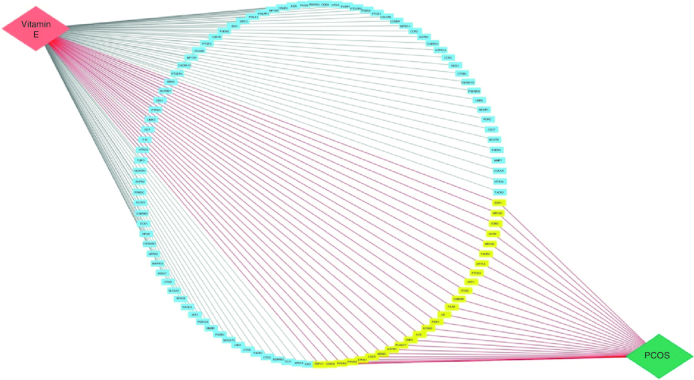
Compound target disease network.

The compound-target-disease (CTD) network diagram, visualizing the relationship between vitamin E and PCOS, was generated using CytoScape 3.9.1. This network consisted of more than 109 nodes connected by 133 edges (interactions) and it exhibited a zero clustering coefficient, as depicted in Figure 6. Within this network, 26 nodes represented the common target genes associated with the drug-disease interaction and these were highlighted. The top ten targets based on topological parameters included *AKT1, PTGS2, PPARG, PPARA, ERS1, LDLR, GSK3B, CNR1, ACE* and *ESR2*. These genes are considered as potential targets for vitamin E in the context of PCOS treatment.

### *In silico* molecular docking

We conducted an extensive exploration through molecular docking simulations to evaluate vitamin E's capability to inhibit various targets linked to hormonal, oxidative stress and inflammatory pathways related to PCOS progression. The calculated binding strengths for all the suggested targets fell within a range from -5.3 to -9.3 kcal/mol (Table 2). While some receptors displayed more robust binding affinities, suggesting strong hydrogen bond interactions, only a specific subset exhibited exceptional biochemical interactions within the binding site of the protein target. We employed the Discovery Studio Visualizer software to scrutinize various targets with high binding energy, with the goal of comprehending the molecular interactions and pinpointing the particular amino acid residues responsible for these interactions at the receptor's binding site (Figure 7). Higher binding affinities, ranging from -9.3 to -7.5 kcal/mol, signify a more robust attachment to the target, whereas affinities within the -6.0 to -5.3 kcal/mol range indicate somewhat weaker yet still significant binding. This range allows for a comparison of binding strengths across different receptors. Receptors with more negative values are likely to be more attractive targets for vitamin E. Researchers may give priority to targets with stronger binding affinities for further exploration or experimental validation. The mention of strong hydrogen bond interactions with receptors suggests a specific kind of interaction contributing to the observed binding affinities. Strong hydrogen bonding can improve the stability of the ligand-receptor complex, indicating the potential for effective inhibition. This information assists researchers in evaluating and contrasting the potential efficacy of vitamin E in hindering various molecular targets associated with oxidative stress and inflammatory pathways.

**Table 2.** Interpretation of *in silico* activity of vitamin E on PCOS.

Protein name	PDB ID	Binding energy (Kcal/mol)	Amino acid residues	Interaction
*AKT1*	1UNQ	-5.3	PROA:42, PROA:51, LEUA:52, GLUA:40, LYSA:39, ALAA:50, TYRA:50, TYRA:38, GLNA:47	Van der waals, Pi-Donor Hydrogen Bond, Alkyl
*PTGS2*	5IKQ	-8.5	THRB:207, TYRB:386, ILEB:409, TYRB:405, VALB:296, VALB:448, VALB:445, LEUB:392, GLNB:204, HISB:389, HISB:387, HISB:208, PHEB:211	Van der waals, conventional hydrogen bond, Pi- sigma, Pi-Pi T- shaped, Alkyl, Pi-alkyl
*PPARG*	5FVJ	-9.3	META:329, LEUA:228, ARGA:288, LEUA:333, LEUA:330, SERA:289, META:364, ILEA:281, PHEA:360, LEUA:353, LEUA:356, PHEA:282, ALAA:278, PHEA:363, HISA:449, GLNA:286, LYSA:367, CYSA:285, TYRA:327, ILEA:326, ALAA:292, ILEA:296, PHEA:226, GLUA:295	Van der waals, Pi-cation, Pi-sigma, Alkyl, Pi-Alkyl
*PPARA*	7E5H	-7.8	LYSA:358, ILEA:354, HISA:440, META:355, PHEA:359, LEUA:321, META:325, META:330, ALAA:333, LEUA:344, CYSA:276, THRA:279, VALA:332, ILEA:272, LEUA:247, ILEA:241, CYSA:275, VALA:255, ALAA:255, ALAA:250, GLUA:251, LEUA:247, ILEA:272, SERA:280, ILEA:317, TYRA:314, PHEA:318	Van der waals, Pi-sigma, Alkyl, Pi-alkyl
*ESR1*	3ERD	-7.5	HISA:547, LYSB:520, GLYB:420, ARGA:548, GLUA:523, METB:522, LEUB:544, TYRB:526, ARGB:548, HISB:547. LYSA:520, SERB:527, ILEB:424, METB:427, GLUB:423	
*LDLR*	7NAM	-6.0	PHEA:305, SERA:149, THRA:283, LEUA:105, ALAA:282, SERA:148, GLNA:194, TYRA:191, PROA:280, ASNA:281, PROA:285	Van der waals, conventional hydrogen bond, Alkyl, Pi-alkyl
*GSK3B*	6Y9S	-8.5	VALB263, ASLB:264, GLUA:137, PROA:136, ASPB:260, ILEA:62, THRA:138, LEUA:188, ALAA:83, LEUA:132, VALA:110, VALA:70, CYSA:199, ASNA:186, ASPA:200, LYSA:85, PHEA:67, GLYA:63, GLNA:185, SERA 261, ARGA:141, LYSA:60	Van der waals, Pi-cation, Alkyl, Pi-alkyl
*CNR1*	8GHV	-6.0	LEUD:252, ALAD:198, SERD:199, VALD:168, TYRD:172, ILEA:169, LYSD:192, LEUD:165, GLYD:195, PHEA:191, TRPD:241, ILED:245, GLYD:194, ALAD:248, VALD:249	Van der waals, Alkyl, Pi-alkyl
*ACE*	3AA1	-8.0	CYSA:354, GLNA:353, ALAA:338, PHEA:511, ASNA:261, TYRA:507, GLNA:265, THRA:364, TYRA:504, GLNA:266, PHEA:441, HISA:367, ASPA:367, ASPA:399, LYSA:438, PHEA:363, SERA:402, LYSA:495, TRA:263, GLUA:150, HISA:337, LEUA:145, CYSA:336, ASPA:146	Van der waals, unfavourable acceptor-acceptor, Pi- anion, Pi- Pi T shaped, Pi-alkyl
*ESR2*	3OLL	-5.4	LEUB:490, PHEB:319, LYSB:314. LETB:494, LEUB:324, GLUB:493, GLUB:332, ALAB:497, VALB:328, GLNB:327, LEUB:331, I;EB:310	Van der waals, Alkyl, Pi-alkyl

Figure 7.Visualizing the molecular docking of vitamin E with PCOS-related targets.A1 and A2 is the 3D and 2D image of AKT1, B1 and B2 is the 3D and 2D image of PTGS2, C1 and C2 is the 3D and 2D image of PPARG, D1 and D2 is the 3D and 2D image of PPARA, E1 and E2 is the 3D and 2D image of ESR1, F1 and F2 is the 3D and 2D image of LDLR, G1 and G2 is the 3D and 2D image of GSK3B, H1 and H2 is the 3D and 2D image of CNR1, I1 and I2 is the 3D and 2D image of ACE and J1 and J2 is the 3D and 2D image of ESR2.

**Figure:**
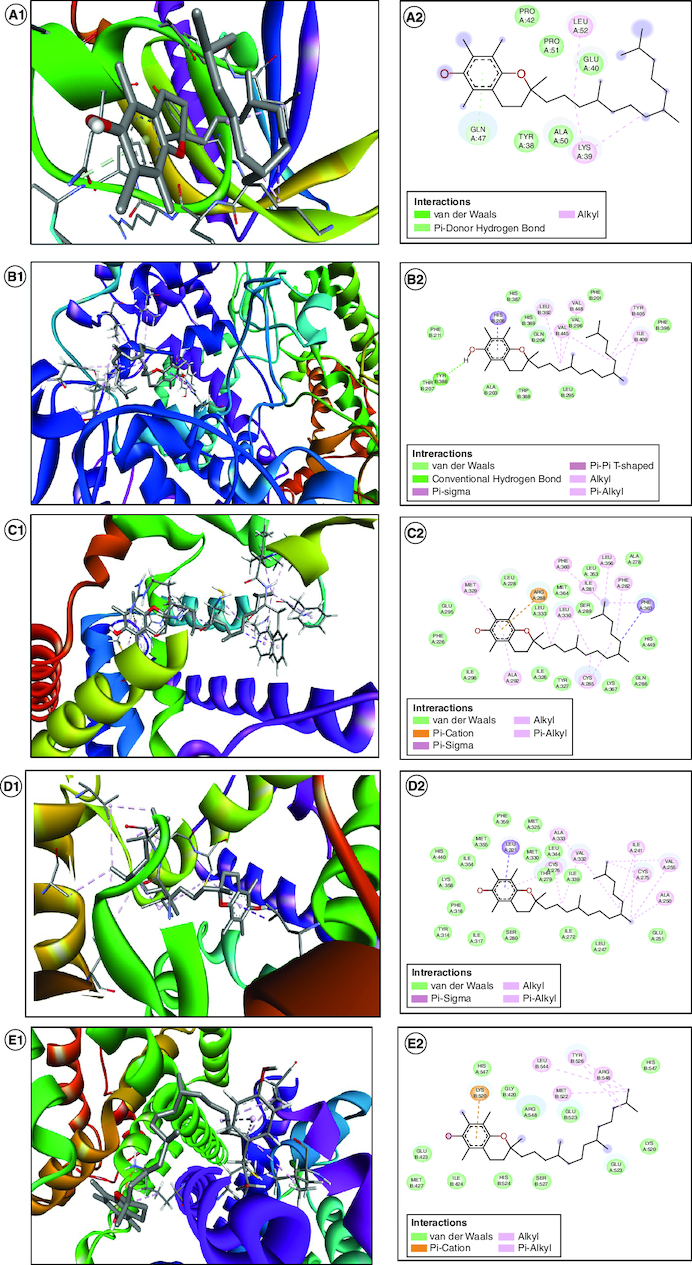

**Figure:**
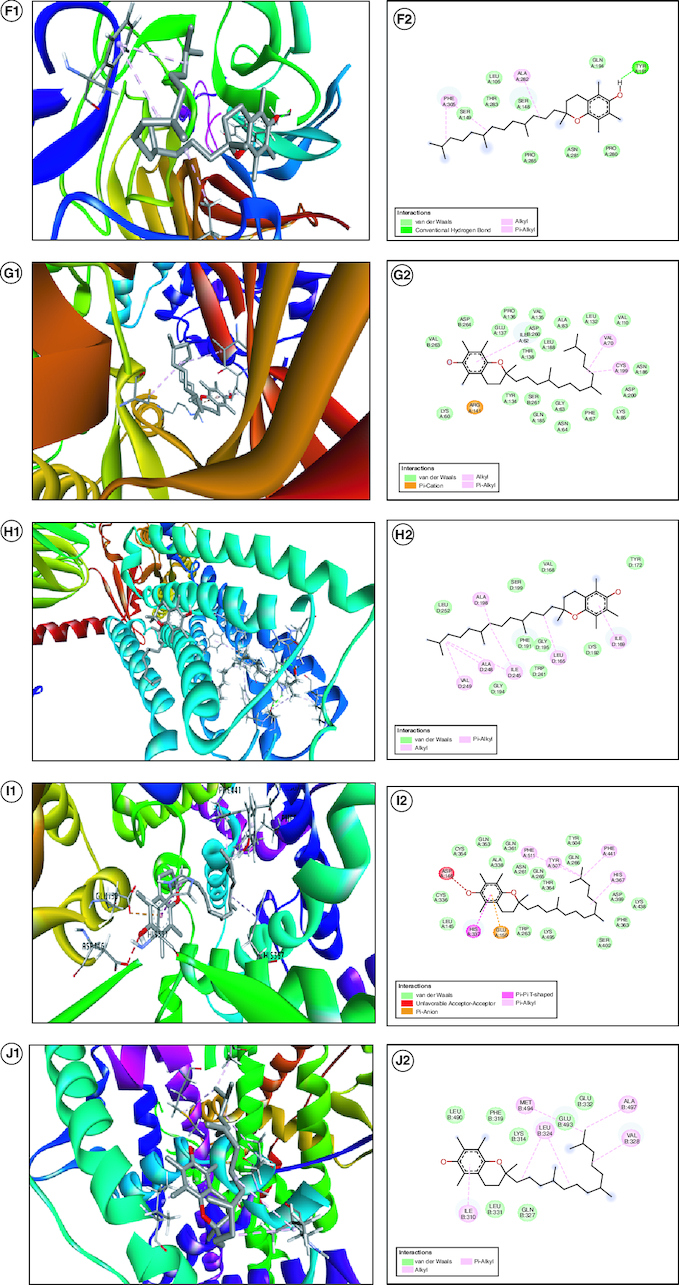

### Interpretation of pharmacokinetic characteristics

The pharmacokinetic profile and predictions related to drug suitability for the synthesized inhibitors, as according on their binding affinities, suggest that the drug with the most favorable binding are promising candidates for drug development. Nonetheless, in the comprehensive procedures of drug formulation, development and exploration, it is imperative to incorporate essential pharmacokinetic features and ADMET considerations as pivotal components. The online tool pkCSM provided a comprehensive analysis of the ADMET parameters for the compound, along with a detailed assessment of its physicochemical properties (Table 3). Subsequently, further scrutiny of the ADMET characteristics (Table 4), such as lipophilicity (Table 5) and water solubility (Table 6), was carried out using the Swiss adme online web tool Lipinski's Rule of Five was utilized to forecast the physicochemical properties essential for a molecule's effectiveness, safety and metabolism [37]. This evaluation revealed variations in log P across three different parameters: Consensus Log Po/w, Log Po/w (WLOGP) and Log Po/w (XLOGP3). The latter three, encompassing atomistic and topological adjustments via Moriguchi's topological method, were employed to assess the logarithm of the partition coefficient within the lipophilicity range. The consensus log Po/w value was derived as an average of the five predictive values mentioned earlier, while SILICOS-IT incorporated a hybrid methodology involving fragments and topological descriptors to estimate the log Po/w. Collectively, these outcomes suggest that all the proposed inhibitors possess pharmacological or drug-like attributes suitable for oral administration.

**Table 3.** Pharmacokinetic profile of Vitamin E.

Parameter	Predicted value
Water solubility (log mol/l)	-6.901
Intestinal absorption (human) (% absorbed)	89.782
P-glycoprotein I/II inhibitor	No/yes
VDss (human) (log L/kg)	0.709
Fraction unbound (human) (Fu)	0
BBB permeability (log BB)	0.876
CNS permeability (log PS)	-1.669
CYP2D6 substrate	No
CYP3A4 inhibitor	Yes
CYP2C19 inhibitor	Yes
CYP1A2/2C9/ 2D6/3A4 inhibitor	No
Total Clearance (log ml/min/kg)	0.794
Max. tolerated dose (human) (log mg/kg/day)	0.775
hERG I/II inhibitor	No/ yes
Oral Rat Acute Toxicity (LD_50_) (mol/kg)	2.072
Oral Rat Chronic Toxicity (LOAEL) (log mg/kg/day)	1.987

**Table 4.** Physicochemical properties of Vitamin E with swiss ADME.

Physicochemical properties	
Parameter	Value
Molecular weight	430.717 g/mol
Num. heavy atoms	31
Num. arom. heavy atoms	6
Fraction Csp3	0.76
Num. rotatable bonds	12
Num. H-bond acceptors	2
Num. H-bond donors	1
Molar refractivity	139.27
TPSA	29.46 Å^2^

**Table 5.** Assessing vitamin E's lipophilicity profile with Swiss ADME.

Lipophilicity	
Parameter	Value
Log *P*_o/w_ (iLOGP)	5.92
Log *P*_o/w_ (XLOGP3)	10.7
Log *P*_o/w_ (WLOGP)	8.84
Log *P*_o/w_ (MLOGP)	6.14
Log *P*_o/w_ (SILICOS-IT)	9.75
Consensus Log *P*_o/w_	8.27

**Table 6.** Assessing vitamin E's Water solubility profile with swiss ADME.

Water solubility	
Parameter	Value
Log *S* (ESOL)	-8.6
Solubility	1.08E-06 mg/ml; 2.50E-09 mol/l
Class	Poorly soluble
Log *S* (Ali)	-11.27
Solubility	2.30E-09 mg/ml; 5.33E-12 mol/l
Class	Insoluble
Log *S* (SILICOS-IT)	-9.16
Solubility	2.97E-07 mg/ml; 6.89E-10 mol/l
Class	Poorly soluble

Despite having a low hydrophilicity score of -6.901, vitamin E exhibits a substantial intestinal absorption rate of approximately 89.782%, making it a promising candidate for oral administration. Additionally, other parameters, including blood–brain barrier (BBB) permeability (log BB) and central nervous system (CNS) permeability (log PS), were found to be 0.876 and -1.669, respectively. These criteria offer understanding into a compound's capacity to navigate biological barriers, yet they constitute merely a single aspect of the drug development journey. Other pivotal elements, including toxicity, metabolism and interactions with targets, also exert substantial influence on a compound's viability as a drug. Furthermore, it's noteworthy that all absorption and permeability parameters align with established threshold values. These results suggest the absence of inhibition of P-glycoprotein II (P-gp), a protein involved in drug transport. The inhibition of P-gp can impact a drug's bioavailability and distribution within the body and while it may be desirable in specific therapeutic contexts, it can also pose clinical challenges during drug development. Additionally, vitamin E was found to be a *CYP3A4* and *CYP2C19* inhibitor, further indicating its effective absorption and significant pharmacological properties [38].

The comprehensive removal of vitamin E from the body is represented by a 0.794 log ml/min/kg clearance value, signifying an efficient elimination process. Notably, the web-based tool indicates that the highest dose of vitamin E that humans can tolerate is 0.775 log m/kg/day and the LD_50_ value, which measures the lethal dose for 50% of the population, is determined to be 2.072 mol/kg. This research underscores the benefits of employing this pharmacokinetic methodology in computational drug development and the analysis of pharmacokinetics. This method produces positive bioactivity scores for potential therapeutic targets.

### Toxicity prediction

Toxicological assessments for the experimental inhibitors in this investigation were conducted through the use of OSIRIS Property Explorer. It is of utmost importance to rigorously scrutinize and validate a drug's toxicity profile before progressing to clinical trials. The toxicity parameters for the compounds examined in this study, as presented in Table 7, revealed that vitamin E exhibited a safe profile, with no indications of mutagenicity, tumorigenicity, irritancy, or adverse effects on reproduction. The majority of the proposed inhibitors demonstrated favorable toxicity profiles, rendering them promising options for treatment. The OSIRIS Property Explorer consistently delivered positive evaluations across all the aforementioned aspects, affirming the safety profile of these compounds.

**Table 7.** Assessing vitamin E's toxicity profile with OSIRIS.

PARAMETERS	Vitamin E (scores)
Mutagenic	GREEN
Tumorigenic	GREEN
Irritant	GREEN
Reproductive effect	GREEN
TPSA	29.46
Drug likeness	-4.78
Drug score	0.12

The Total Polar Surface Area (TPSA) of a molecule holds significant importance in determining its capacity for absorption and tissue penetration. As per the OSIRIS Property Explorer, the Topological Polar Surface Area (TPSA) measurement for the substance was recorded at 29.46. Furthermore, the compound's average drug-likeness score was -4.78. The drug score, which takes into account factors such as molecular weight, toxicity risks and cLogP, is instrumental in assessing whether a chemical compound possesses the potential to meet the criteria for being a viable drug. Vitamin E obtained a drug score of 0.12, indicating its suitability for medical use.

### Pass online web resource

The Prediction of Activity Spectra for Substances (PASS) online tool was employed to anticipate the biological activity of the drugs. This tool projects the range of biological activities of the medications by analyzing their structural formulas, underlining the connection between the chemical structure and their activity [39]. The biological and pharmacological activity of compounds is vital aspects, as they determine the potential applications of these substances in the field of medicine. Furthermore, using the PASS online resource, we also anticipated potential side effects of the medications (Table 8). The PASS online software is employed to assess biologically active substances, providing predictions for over 38 pharmacological actions and biochemical mechanisms, all derived from the structural formula of the compound. These predictions hold promise for the identification of innovative targets with pharmacological actions.

**Table 8.** Prediction of biological activity of vitamin E with PASS online.

Pa (Active Probability)	Pi (Inactive Probability)	Biological activity
0.970	0.002	Lipid peroxidase inhibitor
0.967	0.002	Antioxidant
0.955	0.004	CYP2C12 substrate
0.935	0.004	Acute neurologic disorders treatment
0.932	0.003	Antihypercholesterolemic
0.932	0.005	Antiischemic, cerebral
0.924	0.003	Reductant
0.851	0.002	AR expression inhibitor
0.846	0.005	Hypolipemic
0.842	0.003	Male reproductive disfunction treatment
0.829	0.005	Nucleotide metabolism regulator
0.816	0.002	Prion diseases treatment
0.814	0.006	Antiinflammatory
0.799	0.005	Cholesterol antagonist
0.792	0.002	Anticataract
0.793	0.004	CYP2C19 substrate
0.776	0.005	CYP2E substrate
0.775	0.005	CYP2E1 substrate
0.780	0.011	CYP2C substrate
0.760	0.003	CYP2C8 inhibitor
0.760	0.007	CYP2C8 substrate
0.776	0.041	Ubiquinol-cytochrome-c reductase inhibitor
0.736	0.012	Apoptosis agonist
0.713	0.001	Vitamin
0.704	0.002	Glycogen synthase stimulant
0.709	0.008	Antianginal
0.738	0.043	Testosterone 17beta-dehydrogenase (NADP+) inhibitor
0.712	0.024	TP53 expression enhancer

### Molecular property assessment

The MOLINSPIRATION^®^ software was employed to conduct screening of the selected drugs by in-silico method. Bioactivity ratings for Vitamin E, illustrating its affinity for G protein-coupled receptor (GPCR) ligands, ion channel modulators, kinase inhibitors, nuclear receptor ligands, protease inhibitors and enzyme inhibitors, were assessed using Molinspiration and are outlined in Table 9. The chosen drug was assigned bioactivity rating in diverse categories, including GPCR ligands, ion channel modulators, nuclear receptor ligands, kinase inhibitors and enzyme inhibitors. The bioactivity score was determined based on the compound's binding strength to various receptors, with kinase inhibitors exceeding a bioactivity value of -0.28. The range spanning from -0.50 to 0.00 represents a scale where values closer to -0.50 indicate lower to moderate bioactivity, while values approaching 0.00 suggest a lack of bioactivity or inactivity. A bioactivity score of 0.00 is typically considered neutral, indicating that the compound or drug is inactive. Within this range, values closer to 0.00 imply a decrease in activity as the bioactivity score moves away from -0.50. The assertion that drugs with a score below 0.00 exhibit optimal biological activity suggests that, in this scoring system, lower (more negative) values are associated with higher biological activity. This corresponds with the idea that, in certain cases, compounds with stronger binding affinity or more potent pharmacological effects receive lower scores [40].

**Table 9.** Bioactivity score of Vitamin E using molinspiration.

Parameter	Value
GPCR ligand	0.15
Ion channel modulator	0.07
Kinase inhibitor	-0.28
Nuclear receptor ligand	0.33
Protease inhibitor	0.22
Enzyme inhibitor	0.16

## Discussion

PCOS is most prevalent among women and adolescents of reproductive age, resulting in increased morbidity. It can give rise to various complications, including anovulation, oligoovulation and insulin resistance, ultimately leading to infertility and posing a significant risk to women's reproductive well-being. Unfortunately, a definitive treatment for this condition remains elusive. The exact cause of PCOS remains unclear, but it is relied on to involve a combination of genetic, hormonal and environmental factors [41]. Existing treatments may not effectively address all the varied symptoms and manifestations of PCOS, ranging from hormonal imbalances to metabolic issues and fertility challenges. Some medications used for PCOS management may have side effects and not all patients tolerate them well. This can impact adherence to treatment plans and overall effectiveness. While some treatments can induce ovulation, they might not adequately address fertility challenges or improve reproductive outcomes for all women with PCOS. The long-term safety of certain PCOS medications, especially with extended use, may raise concerns. Exploring alternative treatments with a favorable safety profile is essential. PCOS is a heterogeneous condition and individual responses to standard treatments can vary significantly. Tailoring treatments to individual patient needs remains a challenge.

The role of vitamin E in the context of PCOS is an area of growing interest and investigation. Vitamin E, a fat-soluble antioxidant, plays a crucial role in protecting cells from oxidative stress, which can lead to inflammation and tissue damage. In PCOS, oxidative stress is thought to be one of the contributing factors to the development of insulin resistance, a key feature of the syndrome [42]. Several studies have explored the potential benefits of vitamin E in managing PCOS. Vitamin E has been investigated for its ability to reduce oxidative stress, improve insulin sensitivity and potentially ameliorate some of the symptoms associated with PCOS, such as irregular menstrual cycles and androgen excess. One of the notable findings in these studies is the potential of vitamin E to mitigate oxidative stress, which may contribute to the underlying mechanisms of insulin resistance and hormonal imbalance in PCOS [43]. By reducing oxidative stress, vitamin E could improve insulin sensitivity, potentially leading to better glycemic control in women with PCOS. Additionally, vitamin E's antioxidant properties may have a positive impact on reproductive health in women with PCOS. Some research suggests that it may help regulate menstrual cycles, promote ovulation and reduce androgen levels. These effects are promising for women with PCOS who often face challenges related to fertility and hormonal imbalances [44,45]. Although vitamin E is generally deemed safe when consumed within recommended dietary limits, an excess intake may result in potential side effects. It's crucial to highlight that the adverse effects mentioned, such as digestive issues like nausea, diarrhea and stomach cramps, as well as impacts on immune function and hormonal balance, are commonly linked to elevated doses of vitamin E, frequently stemming from supplements [46].

Our findings highlights the integration of network pharmacology, ADMET studies and *in silico* molecular docking provides a comprehensive understanding of the pharmacological properties, safety profile and potential therapeutic mechanisms of action of the compound. This multidisciplinary approach aids in the identification of central hub genes, critical biological processes and potential therapeutic targets. In recent years, network pharmacology has emerged as a valuable tool for exploring the intricate molecular mechanisms underlying diseases like PCOS. By leveraging the power of bioinformatics, data mining and network analysis, researchers have aimed to gain a deeper understanding of PCOS at a molecular level and identify potential therapeutic targets.

In this study, we have examined the significance of network pharmacology concerning PCOS. We conducted an analysis of key targets by integrating PPI networks with the CTD network approach to comprehensively understand the interactions between a compound, its target proteins and the biological pathways involved. In the context of exploring vitamin E's potential in alleviating polycystic ovary syndrome (PCOS), this approach can shed light on the molecular mechanisms underlying its therapeutic effects. Identified target proteins are mapped to biological pathways using pathway analysis tools. This step helps elucidate the broader impact of vitamin E on cellular processes and signaling cascades. High-throughput omics data, such as genomics, transcriptomics and proteomics, can be integrated to validate and refine pathway information. This step provides a systems-level understanding of vitamin E's effects.

Notably, our investigation highlighted specific targets, including *AKT1, PTGS2, PPARG, PPARA, ESR1, LDLR, GSK3B, CNR1, ACE* and *ESR2*. Vitamin E was found to exhibit a targeting effect on various proteins, including protein kinase, PPARs and estrogen receptors. These interactions are associated with critical pathways involved in endocrine regulation, ovarian steroidogenesis, insulin resistance and signaling through PPARs, thyroid hormones, prolactin, cortisol and estrogen. The AKT signaling pathway plays a crucial role in various ovarian functions, including folliculogenesis. Additionally, it is involved in mediating cell growth and apoptosis triggered by androgens, making it an attractive target for addressing PCOS Similarly, PPAR has a significant impact on fertility in both men and women, given its expression in the reproductive system, hypothalamus and pituitary gland, which collectively regulate hormonal balance. PPAR plays a role in regulating progesterone secretion while reducing androstenedione and testosterone levels [47,48]. In the context of PCOS, estrogen is a key hormone and its receptors ESR1 and ESR2 are responsible for regulating cyclic gonadotropin release through the hypothalamic–pituitary–ovarian axis. The estrogen pathway is vital in the biosynthesis and metabolism of steroids, insulin function, follicle growth and the maturation and release of oocytes [49,50]. The protective function of vitamin E extends to mitigating oxidative stress by counteracting the impact of free radicals and reactive oxygen species (ROS) generated during cellular processes, potentially preventing degenerative diseases like PCOS and cancer. Vitamin E is recognized for its ability to hinder lipid peroxidation, a chain reaction that produces detrimental lipid hydroperoxides. Network pharmacology studies have indicated its influence on the protein kinase A (PKA) signaling pathway, which can be disrupted by ROS and oxidized lipids, leading to the formation of inflammatory cytokines [51]. Antioxidants, including vitamin E, operate through non-enzymatic mechanisms, effectively neutralizing hydrogen peroxide (H2O2) in cells and converting it into the less reactive superoxide, assisted by other antioxidants such as glutathione, superoxide dismutase (SOD) and catalase [52]. Vitamin E has been reported to modulate signaling pathways associated with inflammation. For instance, it can impact the activity of NF-κB, a transcription factor involved in the expression of pro-inflammatory genes. By regulating NF-κB, vitamin E may downregulate the production of inflammatory mediators [53]. These factors collectively emphasize the potential value of vitamin E in the management of PCOS, especially concerning issues related to ovulation.

These networking pathways was further analysed in *in silico* docking method, in which these top ten protein were docked with vitamin E to study the binding affinity. Surprisingly, the PPAR and estrogen receptors highest docking scores (-9.3/ -7.8/-7.5 Kcal/mol) which showed the potential to provide the biological activity for PCOS. The robust binding interactions identified in the molecular docking investigations indicate that vitamin E could potentially influence crucial proteins associated with PCOS. The identification of amino acid residues involved in binding interactions and the presence of strong hydrogen bond interactions contribute to the understanding of its potential inhibitory effects. These findings highlight the compound's suitability as a therapeutic agent targeting specific proteins and pathways associated with the medical condition under investigation. This paves the way for additional experimental confirmation to establish the impact on hormonal levels and anti-inflammatory characteristics. Further exploration of the compound was carried out by ADMET studies through online web tools such as SwissAdme, OSIRIS, PASS and MOLINSPIRATION including its intestinal absorption rate and the absence of P-gp inhibition, suggest its suitability for oral administration. Additionally, its inhibitory effects on key cytochrome P450 enzymes, such as *CYP3A4* and *CYP2C19*, further emphasize its pharmacological potential.

The study's constraints encompass various aspects. In network pharmacology, the reliance on the integration of diverse data sources introduces a dependency on the quality and comprehensiveness of these datasets and incomplete or biased data may compromise the reliability of network analyses. Moreover, the current knowledge of biological pathways, subject to constant updates, may contain gaps in understanding specific interactions and pathways related to vitamin E in the context of PCOS. The tissue- or context-specific effects of vitamin E may not always be fully captured by network pharmacology, as interactions and pathways can exhibit variations across different biological environments. Docking studies, on the other hand, tend to simplify ligand–protein interactions by assuming rigid structures and disregarding dynamic conformational changes. This simplification might not fully represent the intricate nature of interactions within a biological system. The prediction of accurate binding sites on proteins poses a challenge and inaccuracies in binding site prediction can undermine the reliability of docking results. Additionally, the commonly neglected solvent effects in docking studies, crucial in real biological systems, may lead to deviations from actual binding energies. In the realm of ADME studies, challenges arise in extrapolating findings from one species to another, given the variability in the pharmacokinetics and metabolism of vitamin E among different organisms. *In vitro* ADME studies may not entirely capture the *in vivo* dynamics of vitamin E metabolism and distribution, limiting the generalization of results to real physiological conditions. Furthermore, the formulation of vitamin E supplements plays a significant role in its absorption, distribution, metabolism and excretion and this factor is not always adequately considered in ADME studies.

The combined results from network pharmacology, ADMET studies and molecular docking support the compound's potential as a promising candidate for the treatment of the medical condition. Additional experimental verification and clinical investigations are necessary to validate its effectiveness and safety as a treatment. The utilization of *in silico* computational methods serves as an alternative to animal testing, offering deeper insights into how an organism may react to a chemical stressor [54]. Numerous therapeutic approaches have been proposed, aiming to address multiple proteins associated with disease progression. This underscores the importance of concurrently targeting these proteins to achieve a comprehensive grasp of the disease.

## Conclusion

In conclusion, the integration of *in silico* molecular docking studies and pharmacokinetic assessments provides valuable insights into the potential therapeutic role of Vitamin E in PCOS. The molecular docking simulations have shed light on the plausible interactions between Vitamin E and key molecular targets associated with PCOS, suggesting a potential mechanism of action. Additionally, pharmacokinetic assessments have provided crucial information about the absorption, distribution, metabolism and excretion of Vitamin E, contributing to our understanding of its bioavailability and potential efficacy in PCOS treatment. These computational approaches offer a valuable preliminary framework for further experimental validation and clinical studies. While *in silico* studies provide a theoretical foundation, it is essential to conduct rigorous *in vitro* and *in vivo* experiments to validate the predicted interactions and assess the actual therapeutic benefits of Vitamin E in PCOS.

Moreover, considering the multifaceted nature of PCOS, a holistic approach that combines various treatment modalities and addresses the diverse aspects of the syndrome may be crucial for achieving optimal outcomes. In summary, the amalgamation of *in silico* molecular docking and pharmacokinetic assessments provides a promising avenue for drug discovery and development in PCOS, with Vitamin E emerging as a potential candidate worthy of further exploration. Continued research endeavors are imperative to bridge the gap between computational predictions and clinical applications, ultimately advancing our understanding of PCOS pathophysiology and paving the way for more effective therapeutic interventions. Although these discoveries are promising, it is crucial to acknowledge that the utilization of vitamin E at its currently available doses (200 and 400 mg) as a therapeutic strategy for PCOS is an area of research that is still in the process of development. Further research, including well-designed clinical trials, is needed to establish its effectiveness and safety as part of PCOS management.
